# Investigating How Clinicians Form Trust in an AI-Based Mental Health Model: Qualitative Case Study

**DOI:** 10.2196/79658

**Published:** 2025-12-19

**Authors:** Anthony Kelly, Niharika Bhardwaj, Trine Theresa Holmberg Sainte-Marie, Pepijn Van de Ven, Ruth Melia, John Eustis Williams, Kim Mathiasen, Amalie Søgaard Nielsen

**Affiliations:** 1 Department of Electronic and Computer Engineering, University of Limerick, Casteltroy, Limerick, V94 T9PX, Ireland, 353 61 202700; 2 Health Research Institute, University of Limerick, Castletroy, Limerick, Ireland; 3 Department of Psychology and Behavioural Sciences, Aarhus University, Aarhus, Denmark; 4 Department of Psychology, University of Limerick, Limerick, Ireland; 5 Center for Digital Psykiatri, Forskningsenhed, Denmark; 6 Research Unit of Digital Psychiatry, Department of Clinical Research, University of Southern Denmark, Odense, Denmark

**Keywords:** artificial intelligence, AI, explainable AI, XAI, trust, machine learning, ML, mental health, decision support system, intrinsic trust

## Abstract

**Background:**

Trust in artificial intelligence (AI) remains a critical barrier to the adoption of AI in mental health care. This study explores the formation of trust in an AI mental health model and its human-computer interface among clinicians at a web-based mental health clinic in the Region of Southern Denmark with national coverage.

**Objective:**

This study aims to explore clinicians’ perspectives on how trust is built in the context of an AI-supported mental health screening model and to identify the factors that influence this process.

**Methods:**

This was a qualitative case study using semistructured interviews with clinicians involved in the pilot of a mental health AI model. Thematic analysis was used to identify key factors contributing to trust formation.

**Results:**

Clinicians’ initial attitudes toward AI were shaped by prior positive experiences with AI and their perception of AI’s potential to reduce cognitive load in routine screening. Trust development followed a sequential pattern resembling a “trust journey”: (1) sense-making, (2) risk appraisal, and (3) conditional decision to rely. Trust formation was supported by the explainability of the model, particularly through (1) visualization of confidence and uncertainty through violin plots, aligning with the clinicians’ expectations of decision ambiguity; (2) feature attribution for and against predictions, which mirrored clinical reasoning; and (3) use of pseudo-sumscores in the AI model, which increased interpretability by presenting explanations in familiar clinical formats. Trust was contextually bounded to low-risk clinical scenarios, such as preinterview patient screening, and contingent on safety protocols (eg, suicide risk flagging). The use of both structured and unstructured patient data was seen as key to expanding trust into more complex clinical contexts. Participants also expressed a need for ongoing evaluation data to reinforce and maintain trust.

**Conclusions:**

Clinicians’ trust in AI tools is contextually and sequentially constructed, influenced by both model performance and alignment with clinical reasoning. Interpretability features were essential in establishing intrinsic trust, particularly when presented in ways that resonate with clinical norms. For broader acceptance and responsible deployment, trust must be supported by rigorous evaluation data and the inclusion of clinically relevant data types in model design.

## Introduction

### Background

Effective and accessible mental health treatment is crucial in addressing the growing global burden of mental health disorders. Untreated mental health disorders not only impact individual well-being but also result in substantial economic and societal strain through lost productivity, increased health care costs, and reduced quality of life. With the economic burden estimated to be US $5 trillion in 2019 [1], the need for timely and appropriate intervention has never been more urgent.

Like many aspects of society, the treatment of mental health disorders stands to be transformed by artificial intelligence (AI). By recognizing complex patterns and analyzing vast amounts of data, AI has the potential to enhance early detection, personalize treatment, and deliver digital therapy at scale [2,3].

AI adoption depends on factors related to trust, such as system reliability, robust performance, and perceived competence, which is especially important in medical [4] and psychological decision making. In mental health, lack of trust is seen as a key barrier to adoption of AI-based decision support systems [5,6]. Explainability is an important factor in fostering trust in AI systems. When users understand why an AI makes certain decisions (explainability) and can clearly comprehend the reasoning behind those explanations (interpretability), they are more likely to adopt AI.

Trust has also been identified as one of the most critical elements of AI adoption in clinical settings. In a scoping review, Hassan, Kushniruk, and Boryck [7] highlight that a lack of trust remains a major barrier to implementation in health care. Transparency, system reliability, and alignment with clinical reasoning are identified as key facilitators. This reinforces the idea that explainability needs to be considered from the perspective of those expected to use the model in practice. Other stakeholders, such as regulators, also play a role in ensuring that digital mental health tools are safe, effective, and trusted through robust regulation and evaluation, which builds public trust [8].

When an AI model predicts a classification with near 100% probability, this implies that the model is near 100% confident that its prediction is correct. As such, the prediction probability is known as the confidence of the prediction [9]. Uncertainty occurs when an AI model is unsure about its confidence prediction, ie, predictive uncertainty [10]. This may occur due to aleatoric uncertainty (uncertainty in the data), or epistemic uncertainty (uncertainty in the model or a shift in the statistical distribution of the data) [11]. The quantification of predictive uncertainty depends on the type of model being evaluated. In neural network models, a single prediction vector is typically output for each data point. The uncertainty is quantified as the deviation of the true-class probability from 100% confidence, measured as the negative log likelihood, or the deviation of all probabilities in the prediction vector, from the true-class probability, measured as the static calibration error or the Brier score. When predictive uncertainty distributions are available, as in Bayesian, Ensemble, or Monte Carlo dropout models, uncertainty manifests itself as the shape of the probability distribution associated with the prediction, with a narrow, peaked distribution indicating certainty, and a wide, flat distribution indicating uncertainty.

Although confidence and uncertainty estimation are considered essential for safe clinical deployment of AI [12], most machine learning methods in the recent medical literature are said to neglect the important issue of model uncertainty [10]. This is an issue, as it is well known that machine learning models can be confidently wrong [13]. In the absence of an uncertainty estimate, such confidently wrong model predictions may undermine end user trust in the model.

Accordingly, there is a need to understand how trust in AI manifests in the interaction between clinical users and an AI system that predicts both treatment probability and uncertainty, in the context of mental health decision support. This includes understanding how such trust affects decisions to use the system, and in which contexts. This qualitative study explores aspects of trust in clinicians’ use of an AI model as a part of a decision support system for treatment prediction.

### Clinician Trust in AI and the Role of Explainability

Although trust is a prerequisite for adoption, it must be appropriate. The review by Asan et al [14] emphasizes reliability, transparency, and alignment with clinical reasoning as central determinants of clinicians’ trust in AI and argues that trust should be appropriate rather than maximized, in order to avoid unwarranted trust. In mental health specifically, the sensitivity of decisions and the potential for harm heighten the need for trustworthy behavior under distribution shift and within workflow constraints.

Explainability is often proposed as the main lever for calibrating trust; however, its effects are context-dependent. The systematic review by Rosenbacke et al [15] reports mixed effects of explainable artificial intelligence (XAI) on clinicians’ trust. Explanations can increase trust when they are clear, concise, and clinically relevant, but they can also have no effect or reduce trust when they are confusing, cognitively heavy, or poorly matched to the task.

Evidence from applied settings shows similar patterns. The empirical study by Wysocki et al [16] examines explainability, utility, and trust in clinical decision making and describes a mixed role for explanations. Benefits include help with sense-making in ambiguous cases and support for less experienced staff, while drawbacks include added interaction effort and the risk of confirmation bias or over-reliance if limitations are not clear. These findings underline the relevance of the design and presentation of explanations in context.

Accordingly, explanation content should be clinically aligned and should be paired with uncertainty communication, such as calibrated confidence or abstention, in order to discourage unwarranted trust [14,15]. Altogether, trust improves when explanations are concise, fit the clinical task, acknowledge limitations, and respect clinicians’ expertise; conversely, trust erodes when explanations are opaque, verbose, or misaligned with workflow [14-16].

### Explainable AI and Trust

XAI refers to the ability of AI systems to provide explanations of their predictions in a way that is understandable and clear [17]. XAI techniques help bridge the gap between AI algorithms and user comprehension. In their transparency and interpretability for understandability framework, Joyce et al [18] describe how XAI relates to the application of AI in mental health. They argue that trust is built when clinicians and patients feel confident that an AI system’s reasoning is transparent and is seen to align with clinical understanding—a concept similar to face validity. Jacovi et al [19] suggest that users tend to trust an AI if its decision-making process aligns with their expectations of rational behavior. Transparency and interpretability are central to the evaluation of such a rationale.

However, the terms surrounding the explainability, interpretability, and transparency of AI models and their operation lack consensus definitions [20,21]. Explainability refers to the ability to understand why a model has made a specific prediction. Interpretability may be thought of as empowering explainability: providing the ability to understand how a prediction was made. Being able to convey the meaning of an AI model’s operation to a human in understandable terms operationalizes the “how” and is therefore an important aspect of interpretability [21]. Interactivity with the AI model and visualization of its operations also play a role in interpretability [22]. Li et al [23] point out that the user interface is a key factor influencing user experience, acting as the main medium through which an AI system communicates its internal reasoning and decision-making process. The way explanations are presented, therefore, plays an important role in how users form trust in the AI.

Transparency, which is related to interpretability, emphasizes a shared, user-centered understanding of the meaning of model features and their operation [19,21]. Transparency implies that the whole model can be understood [20], or in a less strict sense, that the model features and parameters are understandable [17]. In clinical models, transparency implies that the meaning of features has a direct clinical interpretation [18].

Lacking a consensus and in consideration of the various aspects of explainability, involving trust, understanding, confidence, etc, Barredo Arrieta et al [21] suggest that explainability depends on who is required to understand the model, suggesting the following definition: “Given an audience, an explainable Artificial Intelligence is one that produces details or reasons to make its functioning clear or easy to understand.” In a clinical sense, this may be thought of as AI coformulation.

Understandability is used as a proxy for explainability in the transparency and interpretability for understandability framework of XAI [18]. In this framework, transparency and interpretability are the constituent components of understandability (and therefore, explainability). Consistent with the description of those terms above, interpretability is seen as being determined by the model architecture, structure, and how the prediction is presented (similar to the concept of algorithmic transparency); transparency is determined by the features and data used by the model. Taking logistic regression as an example, interpretability comes from the linear model structure and probabilistic presentation of model predictions inherent in logistic regression. The data and features of such a model can be designed so that the meaning of features has a direct clinical interpretation, fulfilling the requirement for transparency.

Jacovi et al [19] frame trust as an explicit contract between the AI and users in a context in which there is a risk to the user if the AI breaks the contract. The contract lays out what the model is being trusted to provide: transparency, predictability, and fairness (for example). Predictability in this context means that model performance is known to the user and accepted. This may involve an overall performance metric, but it may also involve contextual conditionality, implying that the user knows in which context to trust the model performance and in which contexts the model should not be trusted (eg, on certain data subgroups or out-of-domain data). The mechanisms by which an AI model gains a user’s trust can be understood through a differentiation into intrinsic and extrinsic factors. Intrinsic trust is based on the AI’s reasoning process aligning with human expectations (eg, interpretable models, logical decision-making). Therefore, intrinsic trust depends on the user, for example, a clinician, being capable of interpreting the meaning of the features of a transparent clinical model. Extrinsic trust is based on external behavior or evaluations of the model, such as observing consistent past performance or validation of performance reliability. Extrinsic trust is therefore a trust in the model’s evaluation quality. In this way, trust is seen as having both human and quantitative factors, upon which the user judges whether to trust the model or not.

Trust in AI systems often involves a degree of control relinquishment, yet this trust is highly contextual. As Lipton [20] points out, interpretability is not a one-size-fits-all goal, and trust arises not just from transparency but from the alignment of model behavior with user expectations in specific settings. In many cases, users are willing to trust AI when its judgments match their own, whether or not those judgments are objectively correct. That is, both humans and AI may classify a case as a “mistake” or “non-mistake” depending on the category and context, reinforcing or undermining trust.

This context-sensitive nature of trust highlights the importance of aligning AI outputs with human reasoning, not just through outcomes but through explanations. AI models typically associate data features with predictions based on correlations, whereas humans tend to understand scientific models through cause-and-effect relationships. To bridge this conceptual gap, Holzinger et al [24] introduced the concept of causability, defined as the extent to which AI explanations support users in forming causal understanding from inherently correlational outputs.

In the context of AI recommender systems, Shin [25] found that users’ trust increased when they were able to comprehend explanations in causal terms. Rather than offering purely statistical associations (“feature X correlates with outcome Y”), causability emphasizes understanding why such correlations arise. As such, it is considered a prerequisite for effective explainability. A comparable shift can be observed in high-stakes fields such as suicide prevention, where decision-making must be both evidence-informed and explainable to clinicians. In suicide research, for example, there has been a call to move from risk factors to predictive algorithms [26], and clinical guidelines now advocate a shift away from categorical risk classification toward individualized, formulation-based approaches [27,28].

According to Shin [25], trust in AI systems develops through a dual process: a heuristic route, where users form quick judgments based on prior knowledge or biases, and a systematic route, where trust emerges from deeper evaluation, particularly when explanations are clear, meaningful, and causally informative.

Since explainability depends on humans seeking the explanation, the views of users in the clinical context are central. The purpose of this qualitative study is to explore what affects the trust as perceived by clinical users of an AI model aimed at a mental health decision support system. Using thematic analysis, this study explores how users understand the operation of the model, how they interpret its outputs, and what influences their trust. By focusing on user perspectives, it aims to develop a context-sensitive understanding of explainability that is grounded in clinical reasoning and practice.

## Methods

### Study Design and Procedure

The clinical setting for this study was the Internet Psychiatry Clinic, an internet-based cognitive behavioral therapy (iCBT) service operated by the Centre for Digital Psychiatry within the Mental Health Services of the Region of Southern Denmark [29]. This publicly funded, free-of-charge service is integrated into routine mental health care in Denmark. Individuals self-refer and are screened in 2 stages: an initial request form is followed by a clinical assessment conducted by trained psychologists. The screening referred to in this study is the first part of the clinical assessment, with the second part being a clinical interview. Eligibility criteria include being aged 18 years or older and meeting diagnostic criteria for one of the following conditions: mild to moderate depressive disorder, single phobia, social phobia, or panic disorder with or without agoraphobia [30]. The center delivers its iCBT treatment with a nationwide coverage of approximately 4.8 million adults. The AI model and human-computer interface (HCI) used in the moderated cognitive walkthrough is the Probabilistic Integrated Scoring Model (PrISM) originally reported by Kelly et al [31] (further described below), that predicts the probabilities of the 4 available treatment options—depression, panic disorder, social phobia, and specific phobia; based on patient questionnaires in step 1 of the clinical assessment.

In this study, following informed consent, semistructured interviews were conducted with the clinical psychologist participants, guided by the frameworks of perceived trust in AI discussed earlier. The interviews took place in the clinic in Odense, Denmark, over a period of 2 days. Interviews were conducted one-on-one with one participant and one researcher present in the room.

A trust framework-driven interview guide (Multimedia Appendix 1) was used to ensure that key dimensions of trust were systematically explored across all participants, while also allowing space for participants to raise personal or contextual factors that may have influenced their perception of AI [32]. Questions focused on concepts such as explainability, interpretability, confidence, uncertainty, and perceived trust. The flexible nature of the semistructured format made it possible to follow up on unexpected or participant-driven topics that emerged during the discussion.

To support the interviews, a moderated cognitive walkthrough was carried out. During this process, participants interacted with the AI system while responding to questions. The interaction involved navigating synthetic patient records, using the AI to generate treatment predictions, and reviewing both the prediction and its explanation through the user interface. This method allowed for the observation of trust-related responses during actual system use, aiming to minimize the influence of the interview structure on participant behavior, and helped to identify usability issues that may influence trust [33]. The cognitive walkthrough is particularly useful in assessing the user experience and understanding how people cognitively process the interface [34]. In this study, it also served to situate participants’ reflections in a realistic decision-support context, linking system outputs to users’ thought processes in real time.

Individual interviews provided insight into the cognitive, emotional, and experiential factors shaping trust in AI [32], while the cognitive walkthrough allowed for an examination of how perceptions evolved during live interaction with the system so that the interview structure affected behavior as little as possible [34]. Together, these methods enable a deeper exploration of the factors influencing trust, providing both subjective self-reports and objective behavioral data.

This qualitative study used reflexive thematic analysis (RTA) [35,36] as an individual case study [37] to explore participants’ experiences of using an AI-enabled decision support tool in a Danish web-based mental health service. The aim was to investigate how clinicians perceived trust and explainability in the AI model during real-time interaction. Data were generated using think-aloud protocols embedded within the semistructured interviews, allowing participants to verbalize their thoughts and responses as they navigated the AI interface. This approach made it possible to capture both spontaneous reactions and more reflective evaluations of the model’s performance and trustworthiness.

Individual interviews were selected to provide participants with a private and uninterrupted setting to share their views. This was considered particularly important given the sensitivity of topics such as trust and clinical judgment, where participants might feel more comfortable expressing concerns or critiques in a one-on-one context [32]. The interviews were audio recorded consensually, for transcription purposes. The semistructured interview format allowed for flexibility in exploring participants’ responses while ensuring a consistent set of topics related to perceived trust and explainability in AI [38].

The RTA was conducted by 2 researchers independently, with complementary lenses. Reflexively, one of these (AK) brought an AI trust perspective to the analysis. The other (TTHSM) brought a clinical perspective. Our aim was interpretive, focusing on meaning-making rather than reliability metrics. Consistent with RTA, we did not seek intercoder agreement, did not hold intercoder dialogue aimed at consensus, and did not conduct member-checking. The analysis followed Braun and Clarke’s [35] six-phase process: (1) familiarization with the data, (2) initial coding, (3) generating initial themes, (4) reviewing themes, (5) defining and naming themes, and (6) producing the report. The analysis was conducted as an iterative, reflexive, and nonlinear process, in line with Braun and Clarke’s [36] guidance for RTA [39]. This involved the formulation of semantic codes of surface meaning, but also where an underlying meaning emerged, latent codes were formulated, involving a more subjective interpretation.

### AI Model

**Figure 1.**
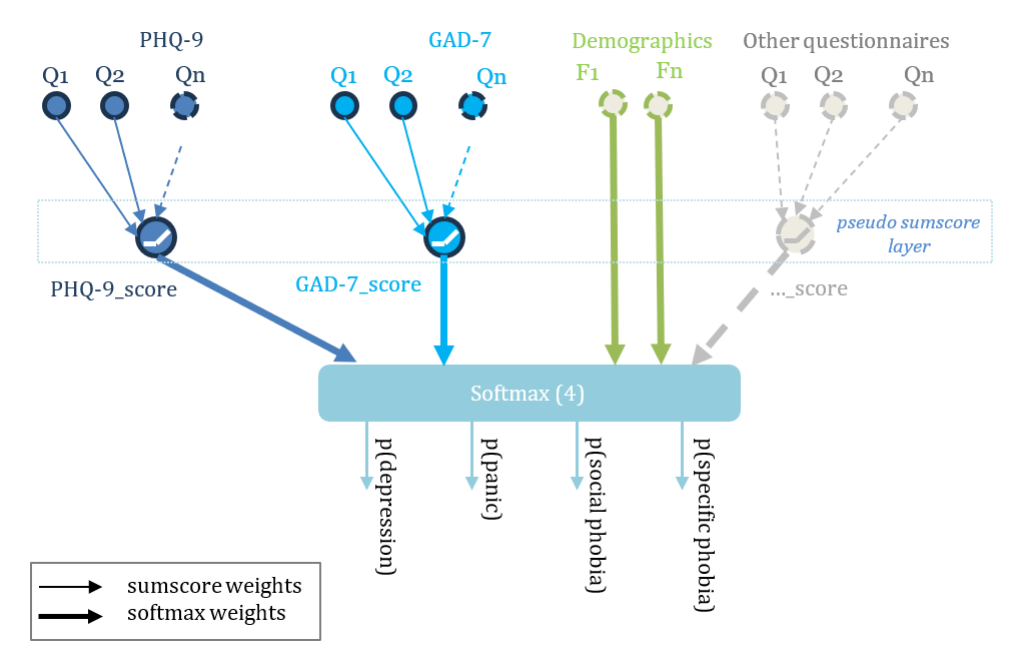
Structure of the Probabilistic Integrated Scoring Model (PrISM). GAD-7: Generalized Anxiety Disorder Questionnaire-7; PHQ-9: Patient Health Questionnaire-9.

The PrISM AI model [31] (Figure 1), used in the moderated cognitive walkthrough, is a 2-level feed-forward neural network that emulates the traditional “sum-score” logic of mental-health screening instruments while retaining full differentiability and probabilistic predictions. Item-level responses from 6 validated questionnaires—Patient Health Questionnaire-9 (PHQ-9) [40], the Generalized Anxiety Disorder Questionnaire-7 [41], the Social Interaction Anxiety Scale (SIAS) [42], the Panic Disorder Severity Scale [43], and the Fear Questionnaire [44], and selected MANSA items—together with 8 sociodemographic variables constitute the input vector. In the pseudosum layer, each questionnaire’s items are combined by nonnegative, L1-regularized weights and passed through a rectified-linear activation, yielding learned questionnaire scores that remain directly interpretable to clinicians. These scores, augmented by the raw demographic features via a skip connection, are then supplied to a softmax output layer that performs multiclass logistic regression across 4 treatment categories (depression, panic disorder, social phobia, and specific phobia).

Uncertainty was quantified at inference time through Monte Carlo dropout: a 5% dropout mask is retained during every forward pass, and the network is sampled 100 times, producing an empirical posterior distribution over class probabilities. This procedure approximates a fully Bayesian logistic model while incurring negligible computational overhead and enables case-level reporting of both confidence and epistemic uncertainty.

**Figure 2.**
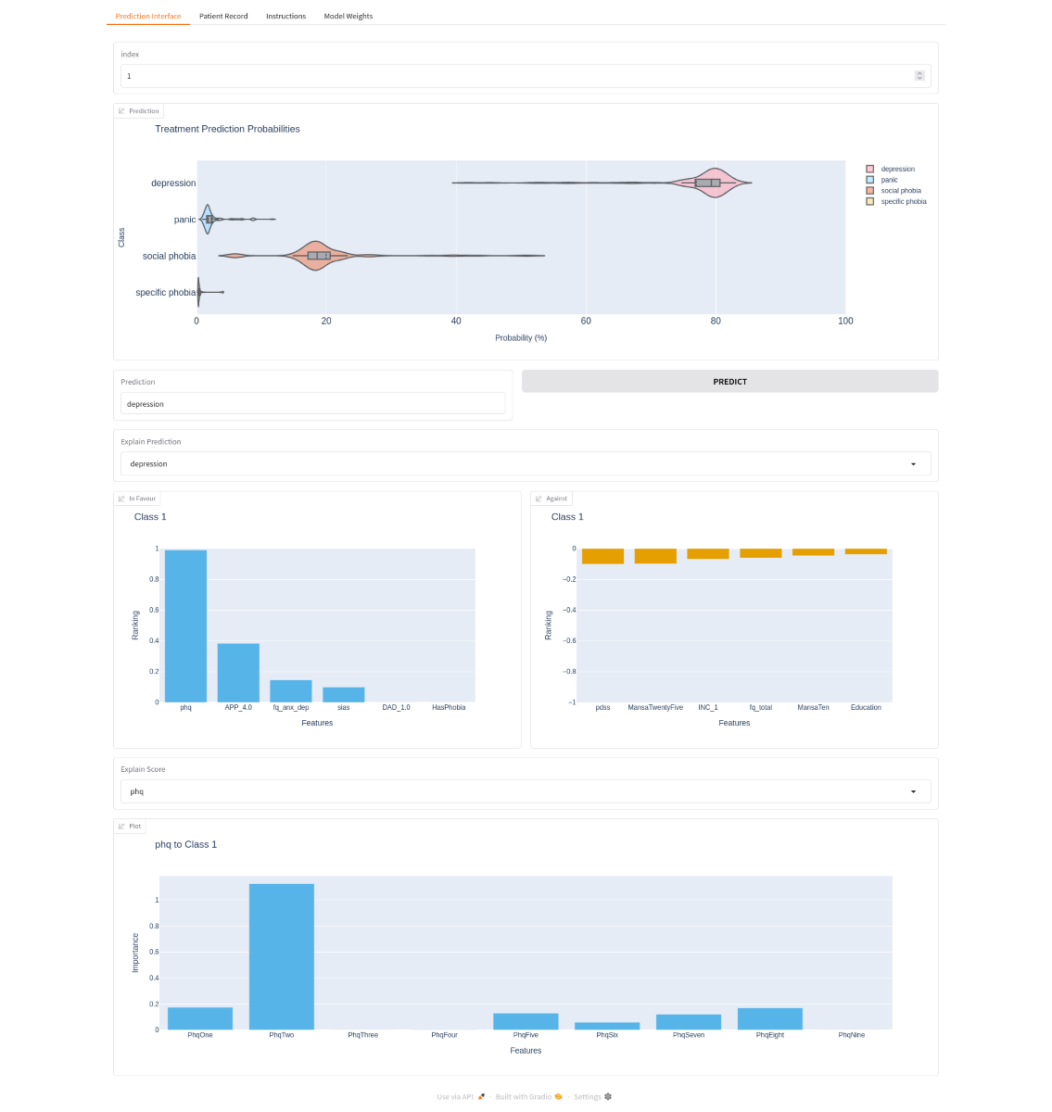
Illustration of the artificial intelligence human-computer interface. The top plot shows the prediction of the 4 classes (y-axis), with the probability distribution of the predictions on the x-axis, illustrated as violin plots. The middle plots show the features that explain the class prediction selected (left) and the features that counter that prediction (right). The bottom plot shows individual questionnaire answers that relate to a selected questionnaire.

The user interface to the PrISM model, presented to the participants, is shown in Figure 2. The top panel shows violin plots of the posterior distribution over class probabilities for the treatment prediction for a specific patient example, indicating a prediction of depression. The explanation of the prediction is shown in the middle panel. This shows graphs of ranked feature importance; the features that explain the class prediction selected (left) and the features that counter that prediction (right). The “explain prediction” dropdown above the panel allows the user to select which of the 4 class predictions (depression, panic disorder, social phobia, and specific phobia) to explain. The bottom plot shows individual questionnaire answers that relate to a selected questionnaire.

**Figure 3.**
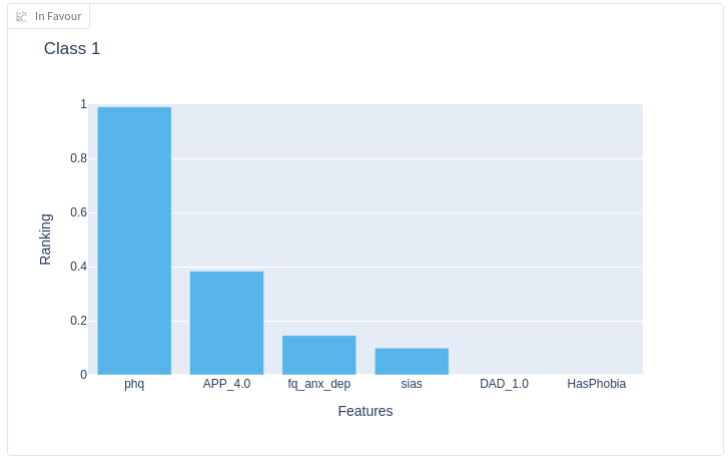
Details of the features that explain the class prediction selected (left middle panel of the artificial intelligence human-computer interface).

The detail of the prediction explanation for a specific patient is shown in further detail in Figure 3. The chart indicates that the PHQ-9 score (phq), the demographic feature “doesn’t know what treatment to apply for” (APP_4.0), and the fear questionnaire anxiety-depression subscale (fq_anx_dep) have the most influence on the prediction of the depression class (class 1) in this specific patient example. When interacting with the AI HCI, hover-overs provided text explaining the meaning of the features and score totals where relevant, for improved usability.

The reference implementation (TensorFlow 2.19, Python 3.11) was trained on retrospectively collected data from 1068 adults referred to the Danish national internet-psychiatry service. Records were stratified by diagnosis and partitioned 75%/25% into development and held-out test sets. Optimization used the Adam algorithm (batch=32, learning rate=0.001) for 2000 epochs, with L1-regularization (λ=0.001) promoting sparsity and retaining only clinically salient inputs.

On the unseen test cohort, the model achieved a balanced accuracy of 0.79 and per-class AUC ranging from 0.91 to 0.98. This architecture, therefore, provides two properties considered essential for the safe deployment of machine-learning systems in frontline mental health care: (1) transparent, score-level explanations that align with routine clinical reasoning and (2) probability distributions that facilitate the identification of low-confidence or uncertain cases.

### Data

A dataset comprising patient questionnaire answers and demographics was used to train the PrISM AI model used in the cognitive walkthrough semistructured interviews. The data were gathered between November 14, 2019, and December 31, 2022, from patients of the “Internetpsykiatrien” web-based treatment services delivered by the Centre for Digital Psychiatry, Denmark. The center delivers routine care and internet-delivered cognitive behavioral therapy with nationwide coverage [29]. The data consisted of answers to the PHQ-9, the Generalized Anxiety Disorder Questionnaire-7, the SIAS, the Panic Disorder Severity Scale, and the Fear Questionnaire. Additional features included demographic information and a brief medical history, such as previously diagnosed conditions. The ground truth of the dataset was the choice of treatment selected by the psychologists in the clinic for each patient. Further details can be found in [31].

### Ethical Considerations

Data for the AI model were extracted from Internetpsykiatrien after approval from the Regional Council in Southern Denmark. Since this was a secondary data analysis, separate informed consent was not required. The Regional Committees on Health Research Ethics for Southern Denmark were informed about the study and were provided with the case number S-20232000‐65. In accordance with Danish national ethical guidelines, no additional ethics approval was needed.

Ethical review and approval were not required for the study on human participants in accordance with the local legislation. The patients/participants provided their written informed consent to participate in this study. The study was reported to the Danish Data Protection Agency. Data were anonymized before being provided to the researchers. No compensation was provided.

### Participants

The participants were clinical psychologists working in the Region of Southern Denmark. The group comprised 5 participants (N=5; 2 female and 3 male), with a mean age of 35.8 years. All participants were members of the screening team at an iCBT clinic within the service. Their role involves screening patients for interview prior to possible treatment, as well as conducting clinical interviews as part of the treatment assessment process.

Participants were recruited based on their clinical role in the service, with a range of experience levels represented, from junior to senior staff. The study included 55% (5/9) of the entire screening team, meaning that the majority of clinicians responsible for initial patient triage were included. This provides a strong representation of the perspectives within the clinical setting and enhances the validity of the findings. To preserve anonymity within this small team, specific age and experience ranges are not disclosed.

In a review of sample size requirements for usability studies, Lewis [45] concluded that 5 participants are typically sufficient to reveal around 80% of usability issues. This implies a sample size of 5 is considered appropriate for a qualitative study focused on human-AI interaction. Thematic saturation appeared to be reached, and the inclusion of most of the staff involved in the relevant clinical task provides additional justification for the adequacy of the sample.

### Structured Interview Guide

A structured interview guide was developed to explore participants’ experiences with the AI model and interface. The interview’s semistructured format guided the participants through a process of (1) enquiring about attitudes to AI in mental health care and in their role; (2) a cognitive walkthrough of using the AI system with various patient scenarios; and (3) an in-depth discussion on trust and explainability (Table 1). The guide consisted of open-ended questions designed to elicit detailed responses about usability and trust. Once introduced to the system (Table 1), the Cognitive Walkthrough and Interface Interaction section allowed for an open use of the AI system with the synthetic patient profiles, allowing an observation of natural behavior with the system, in order to minimize the influence of the interview structure on participant behavior. Since the interviews were limited to 1 hour, further follow-up questions were answered in written form. These included the topics: (1) usability and workflow integration, and (2) feedback and improvement.

**Table 1.** Description of the structured interview.

Topic and subtopic	Explanation/example question
Opening and background	
Reconfirm the informed consent	—^a^
Participant demographics	If not collected ahead of time
Initial attitudes toward AI^b^ in health care	When you hear “AI” in the context of patient care, what comes to mind?
Perceived usefulness of AI in health care	Based on your experience, do you think AI could be useful in patient care or treatment decision-making?
Introduction to the AI system	A brief explanation of the system’s function as a treatment recommendation system for mental health and set expectations for the cognitive walkthrough.
Introduction to AI definitions	
Overview of the AI model	
Instructions for cognitive walkthrough	
Cognitive walkthrough and interface interaction	
First impressions	Take a look at the interface. What are your initial impressions?
Clinical assessment	Looking at the patient record, what would your assessment of the treatment recommendation be for this patient?
Locating and understanding the recommendation	Please use the AI tool to make a prediction for the patient.
Interpreting confidence and uncertainty	Do you feel you understand whether the model is “very sure” or “not very sure” about its recommendation?
Accessing and reviewing the explanation	Can you find or access the explanation for why the AI made this recommendation?
Explanation depth and satisfaction	Does the system provide enough detail about how it arrived at its decision?
Trust and ease of use	So far, do you feel comfortable trusting the system’s recommendations? Why or why not?
In-depth discussion on trust and explainability	
Reliability and past evidence	Would it help your trust if you saw data on how often the AI’s predictions were correct in previous cases of this type?
Transparency	How important is it for you to see exactly how the AI weighs different questionnaire scores?
Interpretability	Would it help to understand how this model generally works, beyond just one case?
Interactivity	Do you feel the interface is interactive enough for you to explore the patient record and model explanations?
Fairness	Do you have concerns about the AI being biased or missing certain patient subtleties?
Closing	
Summarize final thoughts and close	What would be your key takeaway from this experience with the AI system?

a Not applicable.

b AI: artificial intelligence.

To clarify the definitions of the terms used in the interviews, the definitions in Table 2 were provided to participants. Although confidence and uncertainty are somewhat ambiguous, we took confidence to be aligned with aleatoric uncertainty and uncertainty to be aligned with epistemic uncertainty.

**Table 2.** Definitions of terms provided to participants.

Term	Description
Explainable (AI^a^)	Relates to understanding “why” AI models make their decisions.
Interpretable (AI)	Understanding “how” an AI model makes its prediction.
Transparent (AI)	How the prediction is made is clear from the model and features.
Confidence	The “probability” of the prediction.
Uncertainty	How “wide” the probability distribution is.

a AI: artificial intelligence.

### Patient Profiles

Four patient profiles were developed to support the moderated cognitive walkthrough and provide a basis for exploration during the interviews. These profiles were designed to represent a diversity of treatment predictions and comorbidities that are typical of treatment decisions routinely seen in the clinic (Table 3). The patient profiles were accompanied by corresponding data suitable for presentation to the AI model. The data involves the answers to patient questionnaires and demographic data for the various patient scenarios [31]. The data was presented to the participants via a patient record view in the AI interface. The written description, provided in Table 3 for context, was not presented until the postinterview debrief.

**Table 3.** Patient profile scenarios.

Scenarios	Patient profile	Clinical rationale
Scenario 1	Jane presents with persistent low mood, a significant decrease in energy levels, and an overwhelming sense of hopelessness. She reports difficulty concentrating at work and has recently been reprimanded for missing deadlines, which has exacerbated feelings of inadequacy. She also experiences intense anxiety in social and professional settings, including meetings and casual social gatherings, which has led her to withdraw from colleagues and friends. Jane’s symptoms began approximately 8 months ago, following a critical performance review at work. Her lack of energy and motivation caused her low work performance, she believes. Initially, she noticed an increased reluctance to speak up in meetings due to fear of judgment. Over time, this fear extended to informal social settings, making her avoid after-work social events and even casual conversations. Concurrently, she began experiencing a pervasive sense of sadness, feeling “empty” most days, with episodes of crying without a clear trigger. She reports difficulty sleeping, often lying awake ruminating over interactions where she feels she may have been perceived as inadequate. She has lost interest in hobbies she once enjoyed, such as painting and attending book club meetings, citing both a lack of motivation and a fear of being judged by others. Previous episodes: Jane recalls experiencing a similar period of low mood and social avoidance during her late teens but did not seek treatment. Family history: A history of depression in her father. No significant medical conditions or history of substance abuse.	Ambivalent case where either depression or social anxiety could be primary. Would tend to treat depression as a priority.
Scenario 2	Susan M, a 34-year-old administrative assistant, presents with a 6-month history of persistent low mood, chronic worry, and reduced functioning. She describes feeling “stuck” and unable to engage fully in her personal and professional life. Her symptoms began following a period of prolonged work-related stress, which has since resolved, but her distress has persisted and gradually intensified. Susan reports a constant sense of unease that permeates most of her day, particularly in the mornings. She wakes up feeling restless and on edge, with racing thoughts about her responsibilities and potential shortcomings. These worries often spiral into feelings of inadequacy, leaving her preoccupied with the fear of disappointing others. Despite recognizing that many of her concerns are unlikely to materialize, she struggles to control or dismiss them. She also reports difficulty concentrating, which she attributes to her mental preoccupation and lack of energy. There is some anxiety and depression history in her family, with her brother having had severe depression at some point and her mother being an anxious and overbearing person.	Primary depression with features of generalized anxiety.
Scenario 3	John D is a 42-year-old accountant who is currently unemployed. He describes experiencing sudden episodes of intense fear, accompanied by physical symptoms such as chest tightness, shortness of breath, sweating, and dizziness. He reports catastrophic thoughts about having a heart attack. These episodes, which John recognizes as panic attacks, occur unpredictably, often when he is driving or in crowded spaces like grocery stores. The fear of these episodes has led him to limit his outings and rely increasingly on his wife to run errands, though he continues to apply for jobs where he can work from home. John admits that he has started avoiding situations where he feels escape might be difficult, such as long car rides, crowded events, or unfamiliar environments. While he can still attend essential outings, such as dropping his kids off at school, he often feels on edge and hypervigilant for signs of an impending attack. These restrictions have caused strain in his family life, as he worries about burdening his wife and missing family outings. To cope with his anxiety, John has started drinking small amounts of alcohol in the evenings, which he finds calms his nerves and helps him unwind. He is careful not to exceed the recommended daily intake, consuming 1 to 2 drinks a night, and denies any history of problematic drinking. However, he acknowledges that he relies on this habit to manage his discomfort and worries about its long-term implications. John has experienced a general sense of fatigue and low mood. While he denies feeling hopeless or experiencing anhedonia, he describes difficulty finding joy in his usual hobbies, such as woodworking and jogging, because he feels “too keyed up” or “too drained” to engage. His sleep is often restless, disrupted by worry about his health and family, though he sleeps sufficiently most nights. He also reports a pervasive sense of guilt, believing that his anxiety is a weakness and that he is failing to be a strong role model for his children. When John went to the gymnasium, he received a diagnosis of anxiety, but he doesn’t remember much about that, and he did not receive treatment other than antianxiety medication.	Panic disorder.
Scenario 4	Mark, a 37-year-old software developer, presents with a persistent fear of bridges and heights that has mildly impacted his daily life for the past several years. His fear began in his late twenties after a stressful experience driving across a high suspension bridge during a storm, which left him feeling uneasy and lightheaded. While he did not experience a full-blown panic attack, the incident caused him to develop a wariness of high bridges. Over time, this fear generalized to other situations involving heights, such as tall balconies or observation decks. Mark avoids bridges when convenient but does not let his fear entirely dictate his life. He reports experiencing mild physical symptoms, such as increased heart rate and sweating, but these subside once he is off the bridge. Mark also feels discomfort in high places where there is an open or exposed view, such as glass elevators or balconies with low railings. He avoids these situations when possible but can manage if necessary, often relying on grounding techniques like focusing on a fixed point or staying close to a wall. Mark is seeking therapy now because he wants to feel more confident in situations involving heights, particularly as his daughter has expressed interest in visiting landmarks like bridges and observation towers. He is motivated to address his fear so that he can fully participate in family activities without hesitation or discomfort.	Single phobia

## Results

### Thematic Analysis

Consistent with Braun and Clarke’s [36] reflexive approach, we aimed for reflexive interpretive richness, rather than intercoder reliability, with each coder’s distinct point of view contributing to a wider perspective interpreting the data. Therefore, 2 analysts with complementary skills (AK and TTHSM) undertook the RTA, each bringing a distinct epistemic lens.

Coder A, with a nonclinical background in AI trust and HCI, designs and evaluates decision-support tools, holding a pragmatic stance that emphasizes usability, explainability, and uncertainty communication. They adopted a predominantly inductive, bottom-up stance. First, descriptive codes were generated and clustered into provisional subthemes. Since a “trust journey” emerged in the subthemes, a top-down, deductive pass interrogated the data through trust frameworks to articulate the journey in terms of established models. Any latent emerging themes were also preserved.

Coder B, who practices in psychology, prioritizes patient safety, workflow fit, and risk management. They worked with a context-led, primarily semantic focus, ensuring that emergent themes remained grounded in front-line priorities such as areas of clinical hesitancy and reassurance, and how AI intersects with their duty of care.

Braun and Clarke [36] identify this analytic-storytelling mode within reflexive TA when the aim is to surface latent, practice-based logics that a purely code-centric read might miss.

The RTA generated 4 sequential themes. Together they outline a notional “trust journey” that begins with a first encounter and culminates in an informed decision to adopt the tool in real-world screening.

1. “Why engage with AI?”: participants articulate the anticipated utility that motivates engagement.
2. “Making sense of the model”: they attempt to follow and investigate the system’s reasoning, building intrinsic trust and causability.
3. “Should I rely on it?”: they appraised the AI by interpreting probabilistic outputs and explanations in the light of their clinical judgment of the patient record and current work practices.
4. “Deciding to use the model in practice”: they elaborated on when, where, and under what safeguards they would rely on the AI in clinical screening.

**Figure 4.**
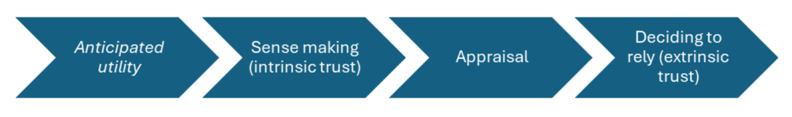
Stages of artificial intelligence–mediated trust.

In Figure 4, the themes are illustrated as a process of cognitive steps in the process of establishing trust:

1. Anticipated utility: “Why engage with AI?”
2. Sense making: “Making sense of the model.”
3. Appraisal: “Should I rely on it?”
4. Deciding to rely: “Deciding to use the model in practice”

In the themes that follow, quotations are referenced by participant ID (P1-P5); square brackets denote clarifications.

### Theme 1: “Why Engage With AI?”—Anticipated Utility

Although none of the clinicians had deployed AI tools in routine practice, four of the five were already experimenting with generative AI in their personal lives. This prior exposure produced a sense of guarded optimism about AI: as one participant put it, in relation to generative AI:

*I don’t necessarily trust the idea it provides; I trust my own interpretation of the idea.*

[P5]

The perceived value of an AI-enabled screening tool is centered around three expectations:

1. Improved efficiency and reduced bureaucracy: Highlighting that AI could examine “Multiple variables at a quick pace—perhaps faster than an experienced clinician could do” (P1); and that screening “Needs to go fast” (P4).
2. Greater consistency and mitigation of human bias: “I think a model like this could maybe also fight...prejudice or bias to some degree” (P2); “I would hope it would be more sure than a person” (P3).
3. Reallocation of clinician time to higher-value tasks: “If it frees me to do more clinical work, that’s very nice” (P2); “Less time looking at numbers” (P5).

Overall, participants framed AI as a complementary aid rather than a replacement for clinical judgment. They were willing to engage with the technology because they believed it could accelerate mundane evaluation tasks, apply rules impartially, and create time for deeper patient engagement. This motivational backdrop set the tone for the subsequent trust-building process described in the following themes.

### Theme 2: “Making Sense of the Model”—Sense-Making Through Intrinsic Trust and Causability

Theme 2 reflects the initial stage of trust-formation where participants inspected violin plots, feature-level importance, and question-level explanations to decide whether the AI’s logic aligned with their mental model of clinical reasoning. Since explanations are only effective if they are understandable to the user [25], this theme captures how participants build intrinsic trust in the AI by attempting to follow and investigate the system’s reasoning.

This theme comprises four subthemes that relate to sense-making and trust-building: (1) orientation and navigation, capturing how participants initially find their way around the interface and data; (2) explainability-by-design, capturing aspects of the model or UI design that improve understanding, currently or in the future; (3) AI-driven insight, capturing clinical or data insights revealed by the model that the user had not initially noticed; (4) UI improvement/usability, capturing improvements that may make the interface easier to use.

During “Orientation and Navigation,” participants navigated the interface easily, spending time on the patient record tab to make their own assessment of the treatment required (as directed in the structured interview). The questionnaire total scores on this tab (eg, PHQ-9, SIAS) were familiar and therefore aided sense-making, often in the form of a patient story:

*And then there’s a graph...And it tells me that there’s...both social phobia and Depression score. There’s symptoms of generalised anxiety and...could be...multiple diagnosis, but it could also be...an image of someone who's depressed and...feels very self-conscious about...dealing with depression. They might be very worried about the future...*

[P2]

However, in the “Explainability-by-Design” subtheme, the y-axis scaling of the bar chart of the questionnaire total scores caused some confusion:

*The bar graph on the scores page may mislead people because that scale has a larger range, it looks like, statistically, it may be much higher, but the range for the depression scale is much smaller.*

[P1]

Other common understandability improvements involved the provision of hover-overs giving more information on the violin plots*:*

*I wish there was some hover over’s here [points at the violin plots].*

[P1]

The graphical explainability, which was enabled by the model transparency, was seen as a major bonus of the AI:

*the graphics impressed me the most, but I think it’s very nice that you can go down and see how the model works*

[P2]

While using the AI model for prediction and subsequently exploring the explanations of the predictions in the interface, some novel insights were revealed, as captured by the “AI-Driven Insight” subtheme. For example, participants found scenario 4 (Table 3) challenging to assess because the questionnaire total scores, which they typically relied on, were uniformly low in this case. The recommended scenario treatment was Specific Phobia, which was explained mainly by the demographic question “Has Phobia.” When the AI prediction explanation revealed this, many participants expressed surprise:

*So, they answered yes. Oh, didn’t see that*

[P3]

*So looking at this...What I'm thinking is I should probably have checked the fear questionnaire for specific phobias, OK, because that's nice.*

[P2]

*So, I will try to explain...to see what it says.[gets explanation from UI] They have actually said that they have a phobia...I think again, it makes sense, if it is...some kind of phobia.*

[P4]

In one case, the prediction in the form of the violin plots, rather than the model explanation, revealed the insight:

*So this one, this definitely helped me this graph [violin plot]...More so than the other ones, because well, because I didn't see the fear...the specific phobia.*

[P1]

As well as highlighting unseen aspects of the patient record, the violin plots also revealed insights about uncertainty, which is typified by this comment from participant 1:

*The probability level wouldn't really matter. It’s just the uncertainty. If they are overlapping too much...definitely it would need to be reviewed by the team.*

[P1]

In the subtheme of “UI Improvement/Usability”, participant 2 saw the Violin plots as the main aspect of sense-making:

*As a Screener I would probably...just look at the photo [violin plots] and then I would...If it wasn’t as clear cut as this. I would click around. But you know it needs to just needs to be easy to work with.*

[P2]

### Theme 3: “Should I Rely on It”—Risk and Uncertainty Appraisal

Since AI adoption depends on user trust [25], theme 3 captured the bridge between making sense of the AI model and deciding to rely on it.

**Figure 5.**
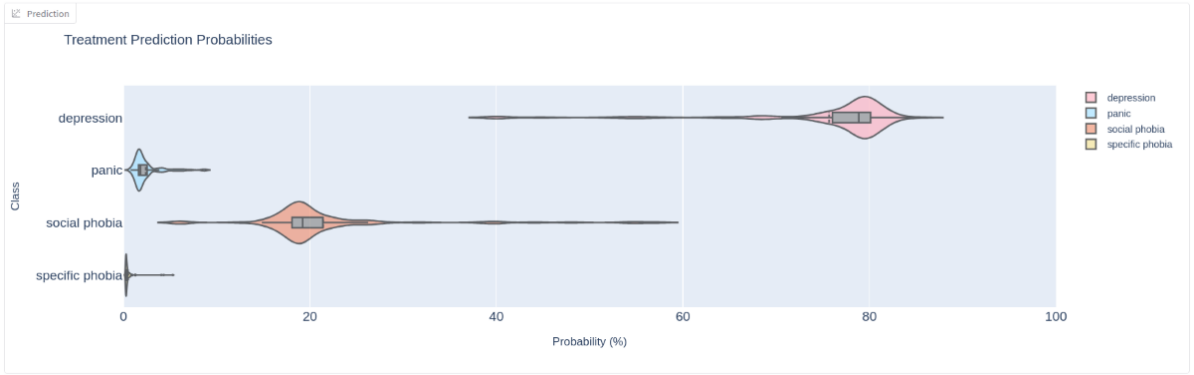
Treatment prediction violin plot for hypothetical patient 1: treatment-depression.

The guided interviews started by defining confidence and uncertainty to set the foundation for the concepts to be discussed. However, the terminology was hard to remember, and the terms were often confused with each other. Nevertheless, participants demonstrated understanding of the concepts from the violin plots, often articulated using their own terms. For example, when referring to confidence, participant 1 says, “Maybe even 70, I think would be sufficient for probability if we don’t have a lot of uncertainty either.” (P1); when referring to uncertainty, participant 3 correctly identifies it as relating to the dispersion of the violin plot, articulating it as being “unsure” “And then social phobia is somewhere between 20% and 40%, but it seems a lot more unsure about what this is actually about” (P3). This shows us that the terminology may be confusing, but the concepts were clear. For example, participants intuitively interpret the violin plots (Figure 5) to reveal the confidence (probability) and uncertainty (dispersion). Participant 4 articulates this well:

*I think I would do it a little bit primitively, just look at where is this big round shape and where is it on the probability percent and yeah, that’s probably how I would do it...And then...about how certain it is? Yeah. OK, so I don’t know if this is very wide or not because I think that this fat bit is quite concentrated...But I think that to me, intuitively I would look at the round bit.*

[P4]

All participants saw the absence of free-text input as a significant limitation of the AI model. In their normal workflow, free text provides significant information:

*looking at what did the person actually write? I think that that’s actually where we get the most information*.

[P2]

Participant 1 stressed that the free text provides important clarifying information over and above the questionnaire scores:

*This...number three here [patient example 3] I think would need to be reviewed because there could be information in the free text that would either clarify this or make us think there’s...it’s too complicated or something else is going on that’s not picked up by our screening measures or the AI prediction.*

[P1]

Although free text was seen as complementary to certain questionnaire items—for example, the Fear questionnaire specific phobia question where the free text describes the specific phobia (P1)—the lack of free-text input was also seen as a limitation to adoption:

*I wouldn’t be comfortable not having the patient’s own words...because it can mean something else.*

[P4]

*Sometimes it can be a very clear-cut depression on the scores alone, but then we read the text, and this is a clear rejection because what they’re applying for is not correct. So, I think if the AI sort of interpret the qualitative text as well and that be even better...I’d be even more in favour of it*

[P5]

### Theme 4: “Deciding to Use the Model”—Extrinsic Trust/Reliance

Theme 4 captures how participants moved from appraising risk to deciding to use the model in practice. In this decision phase, participants looked for external confirmation in the form of agreement with their own judgment, evidence of validation, and consistent performance, before deciding if the AI model could be relied upon, and in what circumstances. Therefore, this theme captured how the AI model fits in the process of patient screening, and the limits of perceived competence of the AI and the data it is trained on.

As patient scenarios were examined, participants demonstrated skill in the use of the AI system: making predictions, drilling down into explanations, then jumping back to the record for demographics. Participant 4 illustrates this fluidity as they gain practice assessing patient scenarios, as though they are conversing with the AI as they think-aloud:

*We start with just panic and say ‘why did you choose this’ and they say that the PDSS score is a significant, which makes sense I think, and also generalised anxiety.*

[P4]

The AI aligning with clinical judgment was an important factor in deciding to trust the AI. Participant 1 provided commentary commensurate with having adopted a trusting attitude whilst staying alert to clinical incongruence:

*I haven’t seen anything to suggest that I would not trust it. Everything fits with my own clinical judgment from what I’ve seen.*

[P1]

Participant 2 highlighted the role of data in trust, implying that an AI system should come to the same conclusions as a person, given the same data:

*[I would trust the AI] To the same degree that I would trust the system we have now because it’s, you know, it’s the same data.*

[P2]

Participant 2 decided to adopt the AI prediction as their own fairly early in the process, when they were somewhat undecided (scenario 1), showing early willingness to put faith in the system. However, in general, trust was seen to be conditional on confidence, certainty, and context. The model could be trusted in screening clear cases to go to an interview, especially for certain cases, but not for making a diagnosis. Participants expressed this as follows:

*If it only comes to screening, then I would probably trust it, especially if it’s certain. And then if it’s not certain, I would maybe spend a little longer to look at it*

[P4]

*For the clear yeses that we don’t have to use our time for that, but they can just go straight to our screening interviews.*

[P3]

*I think the model thinks the same way as a Screener does. But it doesn't think like a clinician does. So as a screening tool, something that might work automatically, I can see how something like this could probably be implemented like soon. As clinical tool, I wouldn’t...with what I see now, trust it to the degree that it could make a diagnosis.*

[P2]

Contextually, participants wanted reassurance that the AI would take into account patient risk factors before they would recommend the AI as a screening tool.

*We cannot have someone with a high suicidal risk. So, but if I knew this prediction took that into account, then that would be OK.*

[P3]

And then only to allow a patient to proceed to the interview, never to exclude a patient:

*I think that’s long as it confirms and does not reject, I think ethically I’m less hesitant...I think as soon as we start rejecting people or limiting people’s access to treatment based on automatic AI responses. That would be problematic.*

[P5]

Screeners routinely assess the severity of presentation (eg, severe depression), and so any decision to put patients through to interview should take account of severity, as well as prediction confidence and uncertainty: “It doesn’t say the degree of the illness or treatment or disorder.” (P3). In addition, some patients (eg, suicidal or severe cases) should be rejected at the interview stage, and an AI putting patients through to an unnecessary interview could be unethical. This re-emphasizes the need to include free text in the prediction, so any prediction is as complete as possible:

*It’s a waste of patient time if they specifically write about something that in their application that I, after an hour’s interview with them, says we can’t help you because of this. But they already informed me about it, so they wouldn’t have to sit there and open up, and get a rejection if I had just...read that before.*

[P4]

The issue of causability was evident when participants questioned the reasons why certain demographics were associated with prediction explanations. Even though the participants all expressed trust in the AI generally, there was a sense that trust was suspended or withheld when explanations were associative but did not lend themselves easily to a causal explanation:

*Yeah, and I understand that it can’t necessarily explain why the data says it, but the more it can explain why, at least guess why the data says, the better. And that would definitely increase my confidence as well...So, it’s not random, but it doesn’t tell the story why the item is important.*

[P5]

Increased use of the AI tended to build trust further:

*Yeah, but I think I trust it more because I agree with it, or it agrees with me. Yeah, I think because I first looked at it and made my mind up from very limited data, actually...But who knows if I’m wrong and this is wrong as well. But I think I definitely trust it more than now that I’ve interacted a bit more with it, yeah.*

[P4]

Once participants had decided to trust the AI, not agreeing with the AI’s prediction, after some experience with it, evoked a suspension but not a revocation of trust:

*With the specific phobias, there was the factors against, so I might not agree, but that doesn’t make me trust the model less.*

[P2]

The violin plots were seen as effective in communicating predictions, but the AI outputting notifications of when to trust/when not to trust, based on the model’s own uncertainty, was thought to be required for deployment.

There was a view that the AI could be more trustworthy than humans because of its systematic reasoning:

*It concerns me when individual clinicians look at an elevation on that score and interpret it, giving it too much weight, but I don’t have a fear that the algorithm is doing that.*

[P1]

However, since the participants were not given data on the verifiable validity of the model as part of the guided interview process, and although trust was evident intrinsically, future use was conditional on the validity being evidenced extrinsically:

*I don’t know how reliable or valid the model is. I think it would be an improvement of the current system.*

[P2]

*Perhaps we will have the data to support this in the future when the appropriate studies have been completed.*

[P1]

## Discussion

### Thematic Analysis

The cautiously positive initial attitudes toward AI set a positive tone and likely eased movement toward reliance. If the baseline attitude had been skeptical, it is possible that the same interface features might have been interpreted more critically. Concerns about algorithmic bias were muted because the model was trained on the same data the screeners themselves used, and it was viewed as representative of the patient population and therefore acceptable.

As participants worked through the interface, a 4-stage “trust journey” (Figure 4) became apparent, providing a useful support to the literature on AI trust that conceptualizes trust as a staged, context-sensitive process [19,25]. First, participants oriented themselves to the user interface layout and information; next, they made sense of the model’s operation; then they appraised risk and uncertainty. Finally, they decided whether to rely on the output, under what safeguards, and in which contexts. Progress along this trust journey was shaped by 2 elements: how specific visualizations supported sense-making and risk appraisal, and how duty-of-care requirements set boundaries for reliance. It is notable that this journey appeared to emerge in the context of using the AI in a free manner to explore various patient scenarios, rather than being enforced by the interview structure.

Understanding confidence and uncertainty in the predictions was central to trust building, with the violin plots communicating both concepts intuitively. Even though the terminology surrounding these concepts was sometimes confused, the understanding was clearly demonstrated and articulated. In use, violin plots were the main aspect that participants referred to and said they would refer to in the future, given the limited time given to screening. In moving back and forth between record, prediction, and explanation, clinicians repeatedly tested the AI’s reasoning against their own judgment and, in doing so, built intrinsic trust.

The dataset available from the clinic did not include the patients’ free-text inputs. From the clinical safety perspective emphasized by Coder B, trust depended on narrative completeness and safeguarding. The absence of free text limited the assessment of suicidality, comorbidity, and context, which in turn constrained willingness to rely on the tool. Integrating patient free text and providing risk-related cues in summary form would extend the contexts in which reliance is acceptable. Relatedly, questions of causability surfaced when demographic variables appeared in explanation panels: correlations were accepted as “in the data,” but clinicians still sought a story that linked those variables to clinical reasoning. Adopting a pedagogical/causability approach, adding ceteris paribus (“what-if”) sensitivity plots to the interface (eg, partial dependence and individual conditional expectation), could be beneficial. These displays let clinicians vary a single input while holding others constant and observe the corresponding change in the model score, supporting understanding.

Once clinicians felt that the system “thought like a screener,” occasional disagreement with a prediction did not revoke trust outright; rather, it triggered a momentary suspension while they compared the model’s output and explanations with their own assessment. An implication is that early alignment between user judgment and model output may accelerate reliance, but it may also seed confirmation bias. A more skeptical starting stance may have led to alignment being scrutinized more stringently.

Willingness to rely on the AI remained conditional. It was strong when the model expressed high confidence with straightforward cases but tempered in the presence of uncertainty or missing contextual information. Participants indicated that the AI may expedite low-risk referrals, but they resisted allowing it to exclude patients or establish diagnoses without human oversight.

Intrinsic trust accrued through interaction with visualizations and explanations; extrinsic trust depended on evidence of validity and calibration. The reliability analysis presented earlier addresses this requirement and should sit alongside uncertainty displays and clear guardrails as deployment criteria.

The clinical perspective (coder B) clarifies 3 conditions for acceptable use: practice-legible explanations, inclusion of narrative data for risk detection, and strict boundaries where stakes or uncertainty are high.

First, explainability must map to everyday reasoning. Violin plots conveyed the prediction confidence effectively, but transparency had to translate into explanations that clinicians could understand in relation to their practice. The graphical explanations (Figure 3) were also effective in this regard.

Second, trust was built on the belief that the AI model and the clinician relied on the same data and had the same “understanding” of it. Repeatedly, participants returned to the absence of free-text in the patient data, believing that short-form questionnaires alone were insufficient for capturing detail, suicidality, or the subtleties of comorbidity. This concern underlined a priority for clinical safeguarding so that the AI builds trust, rather than eroding it by ignoring critical information.

Third, automation boundaries should reflect accountability. The model should be used to confirm low-risk referrals and escalate ambiguity, not to exclude patients or assign diagnoses without human oversight.

Taken together, the clinical perspective aligns with the main themes that depict a pragmatic balancing of promised efficiency against the need for interpretability, contextual trust, and ethically defensible deployment. Only when these conditions are met can the AI become dependable in the screening process. In settings where clinicians are skeptical about AI, explanations may need to be deeper and more informative, uncertainty cues may need to be more explicit, and evidence of external validation may need to be available up front, to avoid premature dismissal or resistance.

Overall, the findings portray trust as contextual and conditional. Clinicians recognize the efficiency and consistency that AI can bring to mental health screening, but require interpretability, narrative completeness, and contextual safeguards before embracing the tool fully. Trust, in this setting, is a negotiated, context-sensitive stance that evolves as users see the model’s reasoning align, or fail to align, with their own.

### Principal Findings

This qualitative study offers several insights into how clinicians in a web-based mental health clinic in Southern Denmark, with national coverage, formed trust in an AI-mental health model and HCI.

The participants’ initial attitude to AI in mental health was framed by their positive interactions in the past with AI (eg, generative AI), and their view of the possibilities for AI to improve productivity or reduce the burden of low-expertise tasks.

Framed by the anticipated utility of AI, trust developed across a further 3 sequential themes that outlined a notional “trust journey”: sense-making, risk appraisal, and a conditional decision to rely. This reflects the literature on AI trust: moving between (1) building intrinsic trust by understanding the model contextually in relation to potential use cases and user experience; (2) deciding the model can fulfill the trust contract; (3) extrinsic trust based on the functional performance. This “trust journey” provides a useful support to the literature on AI trust that conceptualizes trust as a staged, context-sensitive process.

The explainability of the AI was of particular importance in trust building. This involved several elements. First, displaying prediction confidence and uncertainty in violin plots effectively conveyed the ambiguity inherent in differential clinical decision-making. This was found to be of critical importance in building intrinsic trust, underscoring the role of interpretability in building trust in relation to the expert users’ expectations. Second, the presentation of key features influencing the predicted class—including those supporting and opposing the outcome—allowed users to see that the model reasoned in a manner similar to a clinical screener. This underscores the role of interpretability in allowing an understanding to form, that the model “thought” like a clinical screener. Third, the PriSM model’s feature structure, incorporating pseudo-sumscores, improved interpretability by presenting explanations in familiar terms.

Trust was contextually confined to low-risk areas of clinical practice, such as screening patients prior to interview, and with a guarantee that safety protocols would be incorporated (eg, suicidality indicator checks). Incorporating free-text patient data, which clinicians typically rely on in screening, in addition to tabular data, would increase the contextual area of trust because the model would then have exactly the same data available as the screener.

### Limitations

This study offers a focused and detailed, but necessarily bounded view of clinician trust in an AI model and HCI for mental health treatment prediction. Several constraints should be acknowledged.

The participant pool was deliberately small and clinic-specific: 5 screeners from a single web-based mental health clinic in Southern Denmark, providing national coverage. While this concentration allowed for deep, reflexive engagement with the clinic context—and included the majority of its screeners—it also limits transferability, as attitudes, workflows, and organizational norms may differ in other clinical settings or professional cultures.

The encounters took place with a high-fidelity prototype, not a fully deployed system. Although the interface replicated key functions, it operated on prepared case scenarios under interview conditions. Real-world contingencies—time scarcity, partial records, competing clinical duties—were only indirectly represented. The trust trajectory observed here may evolve when the tool is embedded in day-to-day use.

The think-aloud, semistructured protocol channeled attention toward predefined topics. While this ensured coverage, it may have shaped the sequence of concerns, potentially different from those that would surface in naturalistic use. In particular, this may have influenced the notion of a trust journey that surfaced in the analysis. However, it is believed that the protocol reflected a natural workflow wherein clinicians examine the patient record and assess the treatment required. The inclusion of the AI in this workflow seemed to flow naturally during the interviews, and this gives confidence that the semistructured protocol did not overly influence the results.

The available dataset and prototype excluded free-text input, a feature that clinicians regarded as important for detecting suicidality, subtle comorbidity, and patient voice. As a result, participants evaluated a system that they acknowledged as limited; their conditional trust therefore reflects both the strengths of transparent visualizations and explainability and the recognized absence of narrative data.

No quantitative performance metrics, such as accuracy, calibration, or validation results, were provided during the sessions. Participants formed judgments on the basis of interactive experience alone. The trust documented here thus stands without the extrinsic evidence being available to the participants.

Finally, the thematic account is explicitly reflexive. This approach, building on the expertise of 2 complementary perspectives, prioritizes interpretive richness but also means that alternative analytic lenses might organize the material differently. Because the 2 analysts conducted separate reflexive analyses, some interpretive tensions may remain; we view these as theoretically informative.

### Future Work

Future work should address the concerns of participants in evaluating the AI model’s conditional domain of trust. In the contractual view of trust [19], an AI model should “know what it doesn’t know” and communicate this distinction to the user. The importance of known model uncertainty can be appreciated when the data distribution shifts from the statistical distributions of the training data. During these data distribution shifts, the model’s predictions should be more uncertain. Thus, evaluating confidence-based metrics (eg, negative log likelihood, Brier score, static calibration error) in this scenario is an important next step in answering whether the model’s uncertainty estimates are responsive to a shift even when its class predictions are unaffected, which is a desirable property in high-stakes or safety-critical applications where knowing when not to trust a prediction is as important as the prediction itself.

Once trust has been built, participants expressed a desire that less interaction with the AI would be required, so it could fulfill the participants’ hopes for AI to save time. To fulfill this, we require a method of expressing confidence and uncertainty that does not rely upon inspecting violin plots. To this end, approaches such as conformal prediction should be explored as a principled method to express these factors, which are important for the context of trust. In conformal prediction, conformal sets contain the predicted class labels with a mathematical guarantee such that sets that contain more than one label, for example,{depression, panic}, can be interpreted as being uncertain [46]. Accordingly, the size of the prediction set can drive a traffic-light trust indicator: singleton (green), multilabel (amber), and abstention/empty (red).

The integration of free text in the dataset and as a model feature will be another important next step. Architecting a hybrid model that associates the text with the existing model’s tabular and demographic data would bring closure to the issue of detecting suicidality, subtle comorbidity, and patient voice in the patient record. In addition, the severity of conditions expressed in text may be taken into account. We plan to address this limitation by seeking to add the free-text data to the dataset and building a hybrid model, bridging the text and tabular data into a common feature space for model processing.

### Conclusions

This study supports the literature on AI trust by providing insights into how clinicians form trust in AI tools within the context of web-based mental health care. Trust was not given unconditionally but developed over time through a process of understanding, risk evaluation, and conditional reliance. This journey mirrors the AI trust literature in which AI users approach AI through trust heuristics [25] based on the alignment between the perceived explanations and their mental models [24]; ie, causability. Graphical explanations, especially violin-plot probability distributions, were readily interpreted and became the primary vehicle for intrinsic trust.

The explainability of the AI system played a critical role in this trust journey, particularly when interpretability features matched clinicians’ cognitive models and expectations. Trust was seen as contextual [20]; strongest in low-risk, well-defined contexts, and was contingent on safeguards and transparency.

As such, trust was constrained by three factors with varying emphasis: (1) narrative data completeness (the absence of free-text limited perceived safety), (2) causability (clinicians would like an explanatory story, not correlations alone, but were willing to trust the data); (3) external evidence (formal validation metrics were still expected before full explicit trust would be realized). However, once the contextual risk was appraised, using the AI in the areas of trust was evident, leading to explicit trust building in the screening domain.

For AI systems to gain broader acceptance in mental health practice, they must not only be accurate but also interpretable, context-aware, and subject to ongoing evaluation. Expanding the types of patient data the AI can process—especially free-text notes—could extend the boundaries of trust and enhance clinical adoption.

## Supplementary material

10.2196/79658 Multimedia Appendix 1 Study interview guide.

## Abbreviations

AI: artificial intelligence
HCI: human-computer interface
iCBT: internet-based cognitive behavioral therapy
PHQ-9: Patient Health Questionnaire-9
PrISM: Probabilistic Integrated Scoring Model
RTA: reflexive thematic analysis
SIAS: Social Interaction Anxiety Scale
XAI: explainable artificial intelligence

## References

[1] Arias D, Saxena S, Verguet S (2022). Quantifying the global burden of mental disorders and their economic value. EClinicalMedicine.

[2] Olawade DB, Wada OZ, Odetayo A, David-Olawade AC, Asaolu F, Eberhardt J (2024). Enhancing mental health with artificial intelligence: current trends and future prospects. Journal of Medicine, Surgery, and Public Health.

[3] D’Alfonso S (2020). AI in mental health. Curr Opin Psychol.

[4] Choung H, David P, Ross A (2023). Trust in AI and its role in the acceptance of AI technologies. International Journal of Human–Computer Interaction.

[5] Higgins O, Short BL, Chalup SK, Wilson RL (2023). Artificial intelligence (AI) and machine learning (ML) based decision support systems in mental health: an integrative review. Int J Ment Health Nurs.

[6] Antoniou G, Papadakis E, Baryannis G (2022). Mental health diagnosis: a case for explainable artificial intelligence. Int J Artif Intell Tools.

[7] Hassan M, Kushniruk A, Borycki E (2024). Barriers to and facilitators of artificial intelligence adoption in health care: scoping review. JMIR Hum Factors.

[8] Hopkin G, Branson R, Campbell P (2025). Building robust, proportionate, and timely approaches to regulation and evaluation of digital mental health technologies. Lancet Digit Health.

[9] Baan J, Fernández R, Plank B, Aziz W Interpreting predictive probabilities: model confidence or human label variation?.

[10] Kompa B, Snoek J, Beam AL (2021). Second opinion needed: communicating uncertainty in medical machine learning. NPJ Digit Med.

[11] Malinin A, Gales M Predictive uncertainty estimation via prior networks.

[12] Begoli E, Bhattacharya T, Kusnezov D (2019). The need for uncertainty quantification in machine-assisted medical decision making. Nat Mach Intell.

[13] Zhu F, Zhang XY, Cheng Z, Liu CL (2024). Revisiting confidence estimation: towards reliable failure prediction. IEEE Trans Pattern Anal Mach Intell.

[14] Asan O, Bayrak AE, Choudhury A (2020). Artificial intelligence and human trust in healthcare: focus on clinicians. J Med Internet Res.

[15] Rosenbacke R, Melhus Å, McKee M, Stuckler D (2024). How explainable artificial intelligence can increase or decrease clinicians’ trust in AI applications in health care: systematic review. JMIR AI.

[16] Wysocki O, Davies JK, Vigo M (2023). Assessing the communication gap between AI models and healthcare professionals: explainability, utility and trust in AI-driven clinical decision-making. Artif Intell.

[17] Bernardo V (2023). EDPS TechDispatch on explainable artificial intelligence. Eur Data Prot Superv.

[18] Joyce DW, Kormilitzin A, Smith KA, Cipriani A (2023). Explainable artificial intelligence for mental health through transparency and interpretability for understandability. NPJ Digit Med.

[19] Jacovi A, Marasović A, Miller T, Goldberg Y Formalizing trust in artificial intelligence: prerequisites, causes and goals of human trust in AI.

[20] Lipton ZC (2018). The mythos of model interpretability. Queue.

[21] Barredo Arrieta A, Díaz-Rodríguez N, Del Ser J (2020). Explainable artificial intelligence (XAI): concepts, taxonomies, opportunities and challenges toward responsible AI. Information Fusion.

[22] Ali S, Abuhmed T, El-Sappagh S (2023). Explainable artificial intelligence (XAI): what we know and what is left to attain trustworthy artificial intelligence. Information Fusion.

[23] Li H, Zhang R, Lee YC, Kraut RE, Mohr DC (2023). Systematic review and meta-analysis of AI-based conversational agents for promoting mental health and well-being. NPJ Digit Med.

[24] Holzinger A, Carrington A, Müller H (2020). Measuring the quality of explanations: the system causability scale (SCS). Künstl Intell.

[25] Shin D (2021). The effects of explainability and causability on perception, trust, and acceptance: implications for explainable AI. Int J Hum Comput Stud.

[26] Belsher BE, Smolenski DJ, Pruitt LD (2019). Prediction models for suicide attempts and deaths: a systematic review and simulation. JAMA Psychiatry.

[27] Pisani AR, Murrie DC, Silverman MM (2016). Reformulating suicide risk formulation: from prediction to prevention. Acad Psychiatry.

[28] Staying safe from suicide. NHS England.

[29] Mathiasen K, Riper H, Andersen TE, Roessler KK (2018). Guided internet-based cognitive behavioral therapy for adult depression and anxiety in routine secondary care: observational study. J Med Internet Res.

[30] Tarp K, Nielsen SL, Holmberg TT (2023). Therapist perceptions of the implementation of a new screening procedure using the ItFits-toolkit in an iCBT routine care clinic: a mixed-methods study using the consolidated framework for implementation research. Front Psychiatry.

[31] Kelly A, Jensen EK, Grua EM, Mathiasen K, Van de Ven P (2025). An interpretable model with probabilistic integrated scoring for mental health treatment prediction: design study. JMIR Med Inform.

[32] Bryman A (2016). Social Research Methods.

[33] Hine C (2020). Ethnography for the Internet.

[34] Mahatody T, Sagar M, Kolski C (2010). State of the art on the cognitive walkthrough method, its variants and evolutions. Int J Hum Comput Interact.

[35] Braun V, Clarke V (2006). Using thematic analysis in psychology. Qual Res Psychol.

[36] Braun V, Clarke V (2021). One size fits all? what counts as quality practice in (reflexive) thematic analysis?. Qual Res Psychol.

[37] Yin RK (1994). Introduction: The Case Study as a Research Strategy Case Study Res Des Methods.

[38] Hick A, Ziefle M (2022). A qualitative approach to the public perception of AI. Int J Cybern Inform.

[39] Byrne D (2022). A worked example of Braun and Clarke’s approach to reflexive thematic analysis. Qual Quant.

[40] Kroenke K, Spitzer RL, Williams JBW (2001). The PHQ-9. J Gen Intern Med.

[41] Spitzer RL, Kroenke K, Williams JBW, Löwe B (2006). A brief measure for assessing generalized anxiety disorder: the GAD-7. Arch Intern Med.

[42] Mattick RP, Clarke JC (1998). Development and validation of measures of social phobia scrutiny fear and social interaction anxiety. Behav Res Ther.

[43] Houck PR, Spiegel DA, Shear MK, Rucci P (2002). Reliability of the self-report version of the panic disorder severity scale. Depress Anxiety.

[44] Marks IM, Mathews AM (1979). Brief standard self-rating for phobic patients. Behav Res Ther.

[45] Lewis JR (1994). Sample sizes for usability studies: additional considerations. Hum Factors.

[46] Angelopoulos AN, Bates S (2021). A gentle introduction to conformal prediction and distribution-free uncertainty quantification.

