# Supplementary material for: Investigating How Clinicians Form Trust in an AI-Based Mental Health Model: Qualitative Case Study
Source: JMIR Hum Factors. 2025 Dec 19;12:e79658. doi: 10.2196/79658 (PMC12716233; doi:10.2196/79658)
Supplement: Multimedia Appendix 1 [file humanfactors-v12-e79658-s001.docx]

# Explainability and trust in an AI decision support system for mental health treatment prediction: A HCI study

## *Forklarlighed og tillid til et AI-beslutningsstøttesystem til forudsigelse af mental sundhedsbehandling: En HCI-undersøgelse*

**Definitions:**

| **Explainable (AI):** | Relates to understanding ***why*** AI models make their decisions. |
| --- | --- |
| **Interpretable (AI):** | Understanding ***how*** an AI model makes its prediction. |
| **Transparent (AI):** | How the prediction is made is clear from the model and features. |
| **Confidence:** | The **probability** of the prediction. |
| **Uncertainty:** | How **wide** the probability distribution is. |

**Definitioner:**

| **Explainable (AI):**  **(Kunstig intelligens)** | Relaterer sig til at forstå, **hvorfor** AI-modeller træffer deres beslutninger. |
| --- | --- |
| **Kan fortolkes (AI):** | Forstå, **hvordan** en AI-model laver sin forudsigelse. |
| **Gennemsigtig (AI):** | Hvordan forudsigelsen er lavet, fremgår tydeligt af modellen og funktionerne. |
| **Confidence:** | **Sandsynligheden** for forudsigelsen. |
| **Uncertainty:** | Hvor **bred** sandsynlighedsfordelingen er. |

**1. Opening and Background**

**Purpose**: Say hello, collect basic demographic and clinical background, and gather initial attitudes toward AI.

1. **Informed Consent (Informeret samtykke)**
   - Re-confirm that the participant has given informed consent
     - *Bekræft igen, at deltageren har givet informeret samtykke*
2. **Participant Demographics (Collect ahead of time)**
   - Could you please tell me your age and how many years of clinical experience you have?
   - What is your current role/position in the clinic?
3. **Initial Attitudes Toward AI in Healthcare**
   - When you hear “AI” in the context of patient care, what comes to mind?
     - *Når du hører "AI" eller Kunstig intelligens i forbindelse med patientpleje, hvad kommer du så til at tænke på?*
   - Do you currently use any digital or AI-based tools in your clinical practice? If yes, how? If no, why not?
     - *Bruger du i øjeblikket digitale eller AI-baserede værktøjer i din kliniske praksis? Hvis ja, hvordan? Hvis nej, hvorfor ikke?*
4. **Perceived Usefulness**
   - Based on your experience, do you think AI could be useful in patient care or treatment decision-making? Why / why not?
     - *Baseret på din erfaring, tror du, at AI kan være nyttig i patientpleje eller beslutningstagning ved behandling? Hvorfor/hvorfor ikke?*

**2. Introduction to the Personae AI System**

**Purpose**: Give a brief explanation of the system’s function as a treatment recommendation system for mental health and set expectations for the cognitive walkthrough.

1. **Introduction to AI Definitions**

**Note to Interviewer**: You will show the participant the definitions at the start of the document.

1. **Overview of Personae AI model**
   - The Personae AI system is a machine learning model trained on patient questionnaire and demographic data from the clinic, from 2019 to 2022. It is designed to aid in the screening stage, before interview. It suggests a treatment recommendation (e.g., the most likely treatment category) based on the patient’s questionnaire data and demographics. It can predict four treatment categories: Depression, Panic, Social Phobia, Specific Phobia.
     - *Personae AI-systemet er en maskinlæringsmodel, der er trænet på patientspørgeskema og demografiske data fra klinikken fra 2019 til 2022. Det er designet til at hjælpe i screeningsfasen før interviewet. Den foreslår en behandlingsanbefaling (f.eks. den mest sandsynlige behandlingskategori) baseret på patientens spørgeskemadata og demografi. Det kan forudsige fire behandlingskategorier: Depression, panik, social fobi, specifik fobi.*
   - Let me know your immediate thoughts or questions before we begin.
     - *Fortæl mig dine umiddelbare tanker eller spørgsmål, før vi begynder.*
2. **Instructions for Cognitive Walkthrough**
   - Next, I will ask you to walk through how you would use the interface for a hypothetical patient scenario.
   - As you go through each step, please think aloud and describe what you see, what you’re thinking, and any questions or concerns that arise.

**3. Cognitive Walkthrough & Interface Interaction**

**Purpose**: Observe participants’ interactions with the interface to identify usability issues, barriers to trust, and overall explainability.

**Note to Interviewer**: You will show the participant (3) patient scenarios within the Personae AI interface. Allow them time to explore freely. Prompt them to verbalise their thoughts.

- **First Impressions**
  - Take a look at the interface. What are your initial impressions?
    - *Se på grænsefladen. Hvad er dine første indtryk?*
  - Is it clear what information is being presented and where to begin?
    - *Er det klart, hvilke oplysninger der præsenteres, og hvor man skal starte?*
  - *(Observe how the participant explores the interface.)*
- **Clinical Assessment**
  - Looking at the patient record, what would your assessment of the treatment recommendation be for this patient?
    - *Når du ser på patientjournalen, hvad ville din vurdering af behandlingsanbefalingen være for denne patient?*
  - *(Observe how the participant uses the interface to arrive at the recommendation).*
- **Locating and Understanding the Recommendation**
  - Please use the AI tool to make a prediction for the patient.
    - *Brug AI-værktøjet til at lave en forudsigelse for patienten.*
  - Please find and review the AI’s treatment recommendation.
    - *Find og gennemgå AI'ens behandlingsanbefaling.*
  - How easy or difficult is it to locate the recommendation?
    - *Hvor let eller svært er det at finde anbefalingen?*
  - Does the recommendation itself (e.g., “treatment class: depression”) make sense to you?
    - *Giver anbefalingen i sig selv (f.eks. "behandlingsklasse: depression") mening for dig?*
  - Does the probability plot make sense to you?
    - *Giver sandsynlighedsplottet mening for dig?*
  - *(Observe whether the participant seems satisfied or confused about the recommendation.)*
- **Interpreting Confidence & Uncertainty**
  - The system provides an indication of probability or confidence. Is it clear? Do you find it helpful?
    - *Systemet giver en indikation af sandsynlighed eller tillid. Er det klart? Synes du det er nyttigt?*
  - The system provides an indication of uncertainty. Is it clear? Do you find it helpful?
    - *Systemet giver en indikation af usikkerhed. Er det klart? Synes du det er nyttigt?*
  - Do you feel you understand whether the model is “very sure” or “not very sure” about its recommendation?
    - *Føler du, at du forstår, om modellen er "meget sikker" eller "ikke særlig sikker" på sin anbefaling?*

**Note to Interviewer**: Show the participant a slide of four probability plots: i) confident and certain; ii) confident and uncertain; iii) not confident and certain; iv) not confident and uncertain.

- - Can you match each plot with the description?
    - *Kan du matche hvert plot med beskrivelsen?*
  - *(Observe whether the participant seems satisfied or confused about the model’s confidence and uncertainty.)*


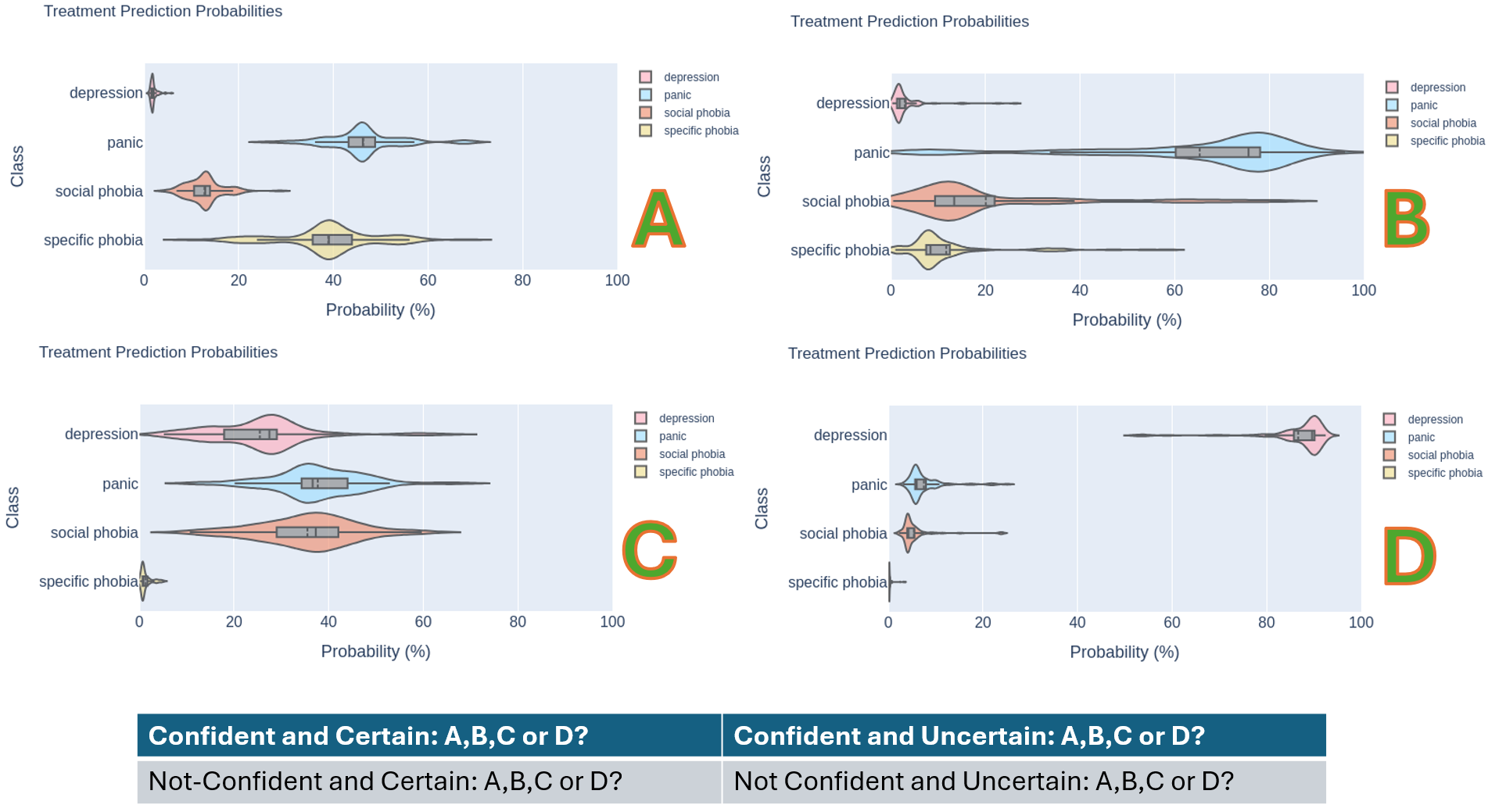


- **Accessing and Reviewing the Explanation**
  - Can you find or access the explanation for *why* the AI made this recommendation?
    - *Kan du finde eller få adgang til forklaringen på, hvorfor AI kom med denne anbefaling?*
  - Is it clear which factors (e.g., questionnaire scores, demographic data) influenced the AI’s suggestion?
    - *Er det klart, hvilke faktorer (f.eks. spørgeskemascorer, demografiske data) der påvirkede AI'ens forslag?*
  - Is it clear which factors supported an alternative suggestion?
    - *Er det klart, hvilke faktorer der støttede et alternativt forslag?*
  - Is it clear which factors mattered more than others?
    - *Er det klart, hvilke faktorer der betød mere end andre?*
  - *(Observe whether the participant seems satisfied or confused about the explanation.)*
- **Note to Interviewer**: Explore all 3 examples (0,1,3).
  - What are the AI recommendations?
    - *Hvad er AI-anbefalingerne for alle eksempler?*
  - Is it clear whether the AI is confident or uncertain in each case?
    - *Er det klart, om AI'en er sikker eller usikker i hvert enkelt tilfælde?*
  - Is the explanation of the recommendation clear in each case (how / why)?
    - *Er forklaringen på anbefalingen klar i hvert enkelt tilfælde (hvordan/hvorfor)?*
  - Looking at the patient record, do you agree with the AI in each case?
    - *Når du ser på patientjournalen, er du så enig med AI i hvert enkelt tilfælde?*
  - *(Observe whether the participant seems satisfied or confused about the prediction and explanations.)*
- **Explanation Depth & Satisfaction**
  - Does the system provide enough detail about *how* it arrived at its decision?
    - *Giver systemet nok detaljer om, hvordan det nåede frem til sin beslutning?*
  - What, if anything, is missing from the explanation that would make you more confident in it?
    - *Hvad, om noget, mangler i forklaringen, der ville gøre dig mere sikker på det?*
- **Trust and Ease of Use**
  - So far, do you feel comfortable trusting the system’s recommendations? Why or why not?
    - *Indtil videre, føler du dig tryg ved at stole på systemets anbefalinger? Hvorfor eller hvorfor ikke?*
  - Would you Trust a model prediction above a certain level of Confidence or Uncertainty to proceed through screening to interview unchecked by a human?
    - *Ville du stole på, at en modelforudsigelse over et vist niveau af tillid eller usikkerhed ville gå gennem screening til interview ukontrolleret af et menneske?*
  - At what level of Confidence or Uncertainty?
    - *På hvilket niveau af tillid eller usikkerhed?*
  - Which elements of the interface or explanation build your trust? Which elements undermine it?
    - *Hvilke elementer i grænsefladen eller forklaringen opbygger din tillid? Hvilke elementer underminerer det?*

**4. In-Depth Discussion on Trust and Explainability**

**Purpose**: Delve deeper into the dimensions of trust—reliability, transparency, interpretability.

1. **Reliability & Past Evidence**
   - Would it help your trust if you saw data on how often the AI’s predictions were correct in previous cases of this type?
     - *Ville det hjælpe din tillid, hvis du så data om, hvor ofte AI'ens forudsigelser var korrekte i tidligere tilfælde af denne type?*
   - Are you interested in seeing a warning if the model is unsure of its prediction?
     - *Er du interesseret i at se en advarsel, hvis modellen er usikker på sin forudsigelse?*
   - How unsure should it be, to issue the warning (in terms of confidence or uncertainty)?
     - *Hvor usikkert skal det være at udstede advarslen (med hensyn til tillid eller usikkerhed)?*
2. **Transparency**
   - How important is it for you to see exactly how the AI weighs different questionnaire scores?
     - *Hvor vigtigt er det for dig at se præcis, hvordan AI'en vejer forskellige spørgeskemascorer?*
   - Does the interface provide enough transparency into the “why” behind the final recommendation?
     - *Giver grænsefladen tilstrækkelig gennemsigtighed i "hvorfor" bag den endelige anbefaling?*
3. **Interpretability**
   - Would it help to understand how this model generally works, beyond just one case?
     - *Ville det hjælpe at forstå, hvordan denne model generelt fungerer, ud over blot ét tilfælde?*
   - Do you find the interface easy to interpret and the results straightforward to apply in a real clinical setting?
     - *Synes du, at grænsefladen er nem at fortolke, og at resultaterne er nemme at anvende i et rigtigt klinisk miljø?*
   - What could be done to make the results more interpretable?
     - *Hvad kan der gøres for at gøre resultaterne mere fortolkelige?*
4. **Interactivity**
   - Do you feel the interface is interactive enough for you to explore the patient record and model explanations.
     - *Føler du, at grænsefladen er interaktiv nok til, at du kan udforske patientjournalen og modelforklaringer.*
5. **Fairness**
   - Do you have concerns about the AI being biased or missing certain patient subtleties?
     - *Er du bekymret for, at AI'en er forudindtaget eller mangler visse patientfinesser?*
   - Do you worry that the AI might systematically misclassify patients with less common presentations or those from different demographic backgrounds (e.g., different age groups, cultural backgrounds, or comorbid conditions)?
     - *Er du bekymret for, at AI systematisk kan fejlklassificere patienter med mindre almindelige præsentationer eller patienter med forskellige demografiske baggrunde (f.eks. forskellige aldersgrupper, kulturelle baggrunde eller komorbide tilstande)?*

- **Note to Interviewer**: These questions may move to follow-up written questions so interview proceeds to Step 7

**5. Usability & Workflow Integration**

**Purpose**: Assess how the system could fit into participants’ usual clinical workflow and how it might affect decision-making.

1. **Workflow Fit**
   - How do you envision using the Personae AI system in your daily routine? (e.g., as a primary tool in screening, a secondary check, a discussion starter, etc.)
     - *Hvordan forestiller du dig at bruge Personae AI-systemet i din daglige rutine? (f.eks. som et primært værktøj i screening, et sekundært tjek, en diskussionsstarter osv.)*
   - What barriers do you foresee in incorporating it into your current workflow?
     - *Hvilke barrierer forudser du for at inkorporere det i din nuværende arbejdsgang?*
   - Would a written report from the Personae AI system be useful? If so, what should be in the report? What should not be in it?
     - *Ville en skriftlig rapport fra Personae AI-systemet være nyttig? Hvis ja, hvad skal der stå i rapporten? Hvad skal der ikke være i det?*
2. **Time & Effort**
   - Does it take too much time or mental effort to interpret the AI’s output?
     - *Tager det for meget tid eller mental indsats at fortolke AI'ens output?*
   - What aspects of the system could be simplified?
     - *Hvilke aspekter af systemet kan forenkles?*
3. **Collaboration Among Colleagues**
   - How do you see yourself discussing the AI’s recommendations with colleagues?
     - *Hvordan ser du dig selv diskutere AI'ens anbefalinger med kolleger?*
   - Would you trust another clinician’s acceptance of the AI’s recommendation without reviewing it yourself?
     - *Ville du stole på en anden klinikers accept af AI'ens anbefaling uden selv at gennemgå den?*

**6. Feedback and Improvement**

**Purpose**: Gather direct suggestions and insights for further development.

1. **Most Valuable Features**
   - Which parts of the interface or explanation are most helpful to you?
     - *Hvilke dele af grænsefladen eller forklaringen er mest nyttige for dig?*
   - Why do you find these features particularly valuable?
     - *Hvorfor finder du disse funktioner særligt værdifulde?*
2. **Areas for Improvement**
   - What would make you more likely to trust or rely on the Personae AI system?
     - *Hvad ville gøre dig mere tilbøjelig til at stole på eller stole på Personae AI-systemet?*
   - Are there additional features or data points you wish the system provided?
     - *Er der yderligere funktioner eller datapunkter, du ønsker, at systemet skal levere?*
   - Do the visual representations (like violin plots) help you interpret the model’s certainty, or are they confusing?
     - *Hjælper de visuelle repræsentationer (som violinplots) dig med at fortolke modellens sikkerhed, eller er de forvirrende?*
   - Do you feel you have enough understanding of how the system was developed overall or which inputs it weighs most heavily in general?
     - *Føler du, at du har nok forståelse for, hvordan systemet blev udviklet generelt, eller hvilke input det vejer tungest generelt?*
3. **Future Use Cases**
   - Under what circumstances would you be most comfortable relying on the AI (e.g., routine cases, borderline cases, complex cases)?
     - *Under hvilke omstændigheder ville du være mest tryg ved at stole på AI (f.eks. rutinemæssige sager, grænsetilfælde, komplekse sager)?*
   - Do you see potential for Personae to be expanded beyond its current functionality?
     - *Ser du potentiale for, at Personae kan udvides ud over dets nuværende funktionalitet?*
   - Do you ever feel that using AI might reduce your professional autonomy or overshadow your own clinical judgment?
     - *Føler du nogensinde, at brugen af AI kan reducere din professionelle autonomi eller overskygge din egen kliniske dømmekraft?*

**7. Closing**

**Purpose**: Wrap up the interview, clarify next steps, and allow any additional comments.

1. **Summarizing Key Points**
   - We’ve covered a range of topics regarding trust, explainability, and usability. Is there anything you’d like to add or emphasize?
     - *Vi har dækket en række emner vedrørende tillid, forklarlighed og brugervenlighed. Er der noget, du gerne vil tilføje eller fremhæve?*
2. **Final Thoughts**
   - What would be your key takeaway from this experience with the Personae AI system?
     - *Hvad ville være din vigtigste takeaway fra denne oplevelse med Personae AI-systemet?*
   - Do you have any final suggestions or concerns?
     - *Har du nogle afsluttende forslag eller bekymringer?*
3. **Thanking the Participant**
   - Thank you for taking the time to participate. Your feedback will directly inform how we refine and improve this system.
     - *Tak fordi du tog dig tid til at deltage. Din feedback vil direkte informere om, hvordan vi forfiner og forbedrer dette system.*

**Instructions for the Interviewer**

- **Ensure Privacy & Comfort**: Begin by emphasizing confidentiality and the voluntary nature of the interview.
- **Encourage Think-Aloud**: During the cognitive walkthrough, remind participants to verbalize their thoughts and feelings.
- **Follow Participant’s Lead**: If they mention an interesting angle or issue, explore it further, even if it’s not on the guide.
- **Maintain Flexibility**: This is a semi-structured guide. Adjust the order or phrasing of questions as needed.
- **Observe Nonverbal Cues**: Note moments of hesitation, frustration, or delight. These can be as telling as verbal responses.
- **Debrief**: After the walkthrough, clarify any points of confusion and thank the participant for their insights.
